# From Traditional Amazon Use to Food Applications: *Tapirira guianensis* Seed Extracts as a Triad of Antiproliferative Effect, Oxidative Defense, and Antimalarial Activity

**DOI:** 10.3390/foods14030467

**Published:** 2025-02-01

**Authors:** Marcell Crispim, Thaise Caputo Silva, Amanda dos Santos Lima, Laura da Silva Cruz, Nathalia Alves Bento, Thiago Mendanha Cruz, Yasmin Stelle, Josiana Moreira Mar, Daniel de Queiroz Rocha, Jaqueline de Araújo Bezerra, Luciana Azevedo

**Affiliations:** 1 Nutritional and Toxicological Analysis Laboratory In Vitro and In Vivo, Federal University of Alfenas, Alfenas 37130-000, MG, Brazil; marcell.crispim@unifal-mg.edu.br (M.C.); thaise.caputo@sou.unifal-mg.edu.br (T.C.S.); amanda.lima@sou.unifal-mg.edu.br (A.d.S.L.); laura.cruz@sou.unifal-mg.edu.br (L.d.S.C.); nathalia.bento@sou.unifal-mg.edu.br (N.A.B.); 2 Department of Chemistry, State University of Ponta Grossa (UEPG), Ponta Grossa 84030-900, PR, Brazil; mcruz.thiago01@gmail.com (T.M.C.); yasminstelle@hotmail.com (Y.S.); 3 Department of Chemistry, Environment, and Food, Federal Institute of Education, Science and Technology of Amazonas, Manaus 69020-120, AM, Brazil; josimoreira54@gmail.com (J.M.M.); daniel.rocha@ifam.edu.br (D.d.Q.R.); jaqueline.araujo@ifam.edu.br (J.d.A.B.)

**Keywords:** erythrocytes, antioxidants, by-product, *Plasmodium falciparum*

## Abstract

*Tapirira guianensis* is a tropical plant found in South America and is widely used by indigenous communities owing to its medicinal properties. Its seeds are rich in phenolic compounds that are known for their anti-inflammatory, antioxidant, and antimicrobial properties. Despite its traditional use, there are limited scientific data on the biological activities of its seed extracts, especially in the context of antimalarial and cytoprotective effects. In this study, we investigated the chemical composition, antioxidant potential, cytotoxic effects, and antimalarial properties of hydroethanolic, ethanolic, and aqueous seed extracts. A 1:1 (*v*/*v*) water/ethanol combination efficiently extracted bioactive compounds and delivered the highest phenolic compound content. Furthermore, the hydroethanolic extracts exhibited significant biological activities, including an ability to reduce cancer-cell viability, protect against damage caused by reactive oxygen species (ROS), and decrease chromosomal aberrations, while exhibiting high efficacy against both chloroquine-sensitive and chloroquine-resistant *Plasmodium falciparum* strains. Hence, the use of *T. guianensis* seed extract as a natural source of bioactive compounds with cytoprotective, antiproliferative, antioxidant, and antimalarial properties is innovative and highlights the need for additional in vivo studies to better elucidate its mechanisms of action and safety.

## 1. Introduction

*Tapirira guianensis* is a non-endemic tree belonging to the Anacardiaceae family and the Tapirira genus and grows in humid soils found in the Amazon rainforest. It is also known in Brazil as “pau-pombo” or “peito-de-pombo” [1]. While the fruit of this tree has traditionally been used for its medicinal properties and economic importance, its chemical and biological potential remain underexplored [2]. Previous studies revealed that *T. guianensis* bark, seed extracts, and juice contain a variety of bioactive metabolites, including phenolic compounds (such as anthocyanins), carotenoids, and others, highlighting its potential antioxidant and antimalarial properties [1,2,3,4]. However, no studies appear to have investigated the cytotoxic effects of *T. guianensis* seed extracts on normal and cancerous cells, or their antioxidant properties toward human red blood cells (erythrocytes).

Oxidative stress, which is driven by an imbalance between the production of reactive oxygen species (ROS) and antioxidant defenses, is a major contributor to the pathogenesis of chronic diseases, such as cancer and malaria [5,6]. The growing global health burden of oxidative stress-related disorders and infectious diseases, such as malaria, and cancer, which are leading causes of morbidity and mortality, necessitate novel therapeutic strategies [7]. Malaria, for example, remains an urgent public health challenge, with approximately 241 million cases and over 600,000 deaths reported annually, predominantly in sub-Saharan Africa [8]. The emergence of drug-resistant strains of *Plasmodium falciparum* has significantly undermined the efficacy of existing antimalarial therapies and highlighted the need for novel targeted chemical compounds [9,10]. Historically, plants have served as sources of antimalarial drugs, such as quinine and artemisinin, which are derived from traditional medicines [11].

Natural products, particularly those rich in phenolic compounds, have gained attention for their ability to mitigate oxidative damage and modulate key biological pathways, thereby offering safer and potentially more effective alternatives to synthetic drugs [12]. *T. guianensis* has been cultivated relatively sparsely; however, its potential applications, including the development of colorless cosmetics and dietary supplements, present promising opportunities for its future advancement [2].

Given the pressing need for new natural sources of bioactive compounds that combat or prevent diseases such as malaria and cancer, and the need to explore the socioeconomic and functional potential of *T. guianensis* using various fruit by-products, this study aimed to explore the chemical composition of *T. guianensis* seed extracts using advanced analytical techniques such as Ultra-Performance Liquid Chromatography coupled with Quadrupole Time-of-Flight Mass Spectrometry (UPLC-Q-TOF-MS). By building on existing knowledge of the plant’s bioactive compounds, we sought to evaluate its potential as a source of therapeutic agents, thereby contributing to the search for innovative and effective treatment options.

## 2. Materials and Methods

### 2.1. Chemical Reagents and Cell Lines

The following reagents were used: ascorbic acid (Vetec, Rio de Janeiro, Brazil), ethanol, sodium hydroxide, aluminum chloride hexahydrate, vanillin (Dinâmica, Indaiatuba, Brazil), Folin-Ciocalteu (Biotec, Curitiba, Brazil), ferric chloride hexahydrate, 2,4,6-tripyridyl-1,3,4-triazine (TPTZ), quercetin, gallic acid, (+)-catechin, chlorogenic acid, sodium nitrite, 2,2-diphenyl-1-picrilhidrazyl (DPPH), (Sigma Aldrich, São Paulo, Brazil), 98% sulfuric acid, 37% hydrochloric acid (Fmaia, Indaiatuba, Brazil), iron (II) sulfate heptahydrate, hydrogen peroxide (Synth, Diadema, Brazil), anhydrous sodium acetate (Anidrol, São Paulo, Brazil), and sodium molybdate (Reatec, Colombo, Brazil). High-Performance Liquid Chromatography (HPLC)-grade methanol, acetonitrile, and formic acid were acquired from Merck (Darmstadt, Germany). Leucine enkephalin reference solution was purchased from Waters Co (Manchester, UK). Other reagents and solvents were of analytical grade and commercially available. Albumax I, Roswell Park Memorial Institute (RPMI)-1640, and Dulbecco’s Modified Eagle’s Medium/Nutrient Mixture F-12 Ham (DMEM/F-12) were acquired from Gibco (New York, NY, USA). 2′,7′-dichlorodihydro-fluorescein diacetate (DCFH-DA), 3-(4,5-dimethylthiazol-2-yl)-2,5-diphenyltetrazolium bromide (MTT), penicillin, Triton X-100, SYBER Gold (S11494), and disodium ethylenediaminetetraacetate dihydrate (EDTA), were purchased from Sigma Aldrich (São Paulo, Brazil).

Human colon carcinoma cells (HCT-8), lung adenocarcinoma cell line (A549), and normal human umbilical vein endothelial (HUVEC) cell lines were obtained from Rio de Janeiro Cell Bank (Rio de Janeiro, Brazil).

### 2.2. Plant Source and Ultrasound-Assisted Solvent Extraction

The fruits of *T. guianensis* were collected from the Manaquiri region (BR319, Km 150) in the Brazilian state of Amazonas, on 14 December 2022. For sample preparation, the fruits were cleaned with 1% sodium hypochlorite solution and the seeds were separated from the fruits for subsequent freeze-drying, resulting in 45 g of sample mass. The bioactive compounds were extracted from the seeds by ultrasound-assisted solvent extraction using a sonicator at 40 Hz and 70 W for 30 min (room temperature) [13]. For each extract, 10 g of dried samples were weighed and processed in a solvent ratio of 1:10 (*m*/*v*), in triplicate. To achieve higher yield, the extraction process was repeated twice, and hydroethanolic, with 50% (HE50) and 80% (HE80) of ethanol, aqueous (AE100), and ethanolic (EE100) extracts were obtained. The ethanolic extracts were dried using a desiccator containing blue silica gel (4–8 mm) that was consistently reactivated in an oven to maintain its drying efficiency. For the aqueous extracts, a lyophilizer (Interprise I lyophilizer, Terroni, Brazil) was employed, operating for approximately 24 h to ensure effective dehydration. Thereafter, the dried extracts were stored at −20 °C until further analysis.

### 2.3. Chemical Profile and Antioxidant Capacity

#### 2.3.1. Phenolic Compounds

The total phenolic content (TPC) of *T. guianensis* seed extracts was determined using the Folin–Ciocalteu assay, and results were expressed as mg of gallic acid equivalent/g of dry matter (mg GAE/g). Different dilutions of *T. guianensis* seed extracts were added to a 96-well plate (25 µL/well), followed by 200 µL of distilled water and 25 µL of Folin–Ciocalteu reagent (1:3, *v*/*v*). After 5 min, 25 µL of 10% sodium carbonate was added, and the absorbance was measured at 725 nm after 1h reaction. A calibration curve (10–80 mg/L, R^2^ = 0.998) was created using gallic acid to compare the absorbance values [14].

The total flavonoid content (TFC) of extracts was estimated using the colorimetric method based on the complexation of aluminum with flavonoids and the results were expressed as mg of catechin equivalent/g (mg CE/g). The total flavonol content was quantified using quercetin as the standard (mg QE/g), and ortho-diphenols were measured through a metallic complex formation with sodium molybdate dihydrate, expressed as mg of chlorogenic acid equivalent (mg CAE/g) [15,16].

#### 2.3.2. Ultra-Performance Liquid Chromatography Coupled with Quadrupole Time-of-Flight Mass Spectrometry (UPLC-QToF/MS) Analyses

The dry extracts (EE100, HE50, HE80, and AE100) were dissolved in methanol and filtered through a polyvinylidene fluoride (PVDF) membrane (13 mm × 0.22 μm, WHATMAN) to a chromatographic vial (1.5 mL). Filtered samples were sonicated to remove air bubbles for analysis using UPLC-QToF/MS analyses. The analyses were performed on a Waters Xevo^®^ G2-XS QTof mass spectrometer (Waters Co., Manchester, UK) coupled to an Acquity HClass UPLC and monitored with MassLynx^®^ software (v. 4.1). Separation of the metabolites was achieved on an Acquity UPLC BEH C18 with a reverse phase column (100 mm × 2.1 mm i.d, 1.7 μm particle size) (Waters, Milford, MA, USA) at 40 °C (±2 °C) and eluted with a gradient system of 0.1% formic acid aqueous solution (A) and 0.1% formic acid in acetonitrile (B) at a flow rate of 0.3 mL/min, using the following linear gradient elution program: A:B, in %: 0–15 min (98:2), 15–20 min (80:20), 20–25 min (60:40), 25–27 min (2:98), 27–27.10 min (98:2), 27.10–30 min (98:2). The injection volume was 10 μL.

The nebulization process operated in negative mode, with a mass range between 100 and 1500 amu, and a scan time of 0.2 s. The Electrospray Ionization (ESI) source parameters were a capillary voltage of 3.0 kV, desolvation temperature of 250 °C; source temperature of 100 °C; cone voltage of 30 V; and cone gas flow of 50 L/h. The desolvation gas flow for negative polarity is 700 L/h. Both low (MS1, 6 eV) and high (MS2, ramped from 20 to 35 eV) collision energy data were recorded by employing MSE continuum mode, an acquisition time of 0 to 30 min, and mass correction during acquisition by an external reference (LockSprayTM). Leucine enkephalin (*m*/*z* 554.2615 [M-H]− and 556.2771 [M+H]+) was used as the lock mass calibrant [17,18].

#### 2.3.3. Nuclear Magnetic Resonance (NMR) Analysis

The dry extract (5 mg) was dissolved in 530 μL of CD3OD containing TMS (≥99.0% purity) as the internal reference (0.0 ppm). This solution was transferred to a 5 mm NMR tube. NMR analyses (1H, 13C, DEPT135, 1H-1H COSY, HSQC, and HMBC) were performed on a 11.7 T spectrometer (Bruker^®^ Avance III HD 500.13 MHz for 1H and 125.8 MHz for 13C, BBFO Plus SmartProbeTM, New York, NY, USA) at 298 K [17,18].

#### 2.3.4. Chemical Antioxidant Capacity

The antioxidant activity of *T. guianensis* seed extracts was evaluated using the following assays: the free radical-scavenging activity against the DPPH radical- and hydroxyl radical-scavenging activity (HRSA) were assessed following an adapted method of Mohammadi et al. and Teng et al. [19,20]. Results were expressed as mg of ascorbic acid equivalent/g of dry matter (mg AAE/g) and mg of gallic acid equivalent/g of dry matter (mg GAE/g), respectively. The ferric-reducing antioxidant power (FRAP) of the extracts was quantified according to Granato et al. [16], with data expressed as mg of ascorbic acid equivalent/g of dry matter (AAE/g).

#### 2.3.5. Lipid Peroxidation Assay

The thiobarbituric reactive substances (TBARS) method was used to evaluate the lipid peroxidation levels in egg yolk, with oxidative stress induced by FeSO_4_ 4 mmol/L solution at 37 °C for 45 min, following the method proposed by Fidelis et al. [21]. The samples were tested at 50 to 250 µg/mL, and the lipid peroxidation inhibition capacity was measured according to the following equation:

lipid peroxidation (%) = (A_Sample_/A_Control_) × 100 (1)

where A_Sample_ was the absorbance at λ = 532 nm of the samples and A_Control_ was the absorbance at λ = 532 nm of the control. All the results were compared with the efficacy of quercetin (5 µg/mL).

### 2.4. Cell Culture: Cytotoxicity and Antioxidant Activity

#### 2.4.1. Cell Viability Assessment

The cytotoxicity of the *T. guianensis* seed extracts was evaluated on HCT-8, A549, and HUVEC cell lines. The cells were cultured in DMEM/F-12, supplemented with 10% fetal bovine serum and 100 μg/mL of penicillin. The cytotoxic activity of samples was observed by a 3-(4,5-dimethylthiazol-2-yl)-2,5-diphenyltetrazolium bromide (MTT) assay [14]. The cells were seeded in 96-well plates at 1 × 10^4^ cells/well (HCT8 and A549) and 6 × 10^3^ (HUVEC), 100 μL/well. The plates were incubated for 24 h at 37 °C and 5% CO_2_ for cell adhesion. After that, the cells were treated with serial concentrations of 10 to 300 μg GAE/mL of extracts and incubated for 48 h. Following the treatment, 10 μL of MTT reagent (0.5 mg/mL) was added to each well and the plates were incubated for 4 h at 37 °C. The formazan crystals were dissolved with 100 μL of dimethyl sulfoxide (DMSO). The absorbance was measured at 570 nm and the 50% cell viability inhibition (IC_50_) was calculated, following the equation

IC_50_ = (T48h/C48h) × 100 = 50 (2)

where T = number of cells after 48 h treatment; C = control cells at 48 h. The Selectivity Index (SI) value was calculated by SI = IC_50_ normal cells/IC_50_ tumor cells.

#### 2.4.2. Intracellular Reactive Oxygen Species (ROS) Generation

ROS generation was assessed using the DCFH-DA assay [16]. Normal (HUVEC) and cancer (A549 and HCT8) cells were placed in a 96-well plate (5 × 10^4^ cells/well) and treated with different concentrations of *T. guianensis* seed extracts (10, 25, 50 μg GAE/mL), which were diluted in DCFH-DA solution (25 mmol/L). The cells were only treated with culture medium for the negative control, and for the positive control, the cells were treated with 15 μmol/L of hydrogen peroxide (H_2_O_2_). The plates were incubated at 37 °C for 1 h in the dark. Following the treatment, the plates were washed with phosphate-buffered saline (PBS), and Hanks’ solution with H_2_O_2_ (15 μmol/L) was added to the wells. The fluorescence intensity (λ emission = 538 nm and λ excitation = 485 nm) was measured, and the data was expressed as the percentage of fluorescence intensity relative to the negative control group.

#### 2.4.3. Protection Against Chromosomal Aberration

The in vitro chromosomal aberration test is used to evaluate the protection or genotoxic effect of substances. The selection of the cell line should mainly concern the culture’s growth ability. A549 cell line and the highest dosages of 50% hydroethanolic extract of *T. guianensis* seed extract were used, based on the results of the cytotoxicity assay. The cells were seeded in 25 cm^2^ flasks at 5 × 10^5^ cells/flask. The positive control was treated with 4 μM cisplatin, a drug that induces chromosomal aberrations, and the negative control received culture medium [22]. The treatment groups received different concentrations of HE50 (5, 10, and 20 μg GAE/mL) in combination with cisplatin (4 μM). Two treatments were prepared to investigate whether the sample can generate genotoxicity independently (20 μg GAE/mL and 50 μg GAE/mL). The flasks were incubated at 37 °C for 48 h, 200 μL of colchicine solution (0.0016%) was added to each group for 6 h, and slides were prepared after fixation and the staining of the material. For the analysis, the chromosomal breakage criteria were used, and the chromosomal aberration rate (%) was calculated as the percentage of chromosome breaks observed per total chromosome.

#### 2.4.4. Erythrocyte Cellular Antioxidant Activity and Protection

To access the interaction of *T. guianensis* and human erythrocytes, oxidation was induced by 2,2′-azobis(2-amidinopropane) dihydrochloride (AAPH), and hemolysis and intracellular ROS generation were evaluated, according to the method described by Cruz et al. [23]. First, fresh blood (O^+^) was obtained from a female volunteer and collected in heparinized tubes after an informed consent form was signed (Federal University of Alfenas-Ethics approval n° 6.910.474). The blood was washed with PBS until it reached 20% hematocrit and mixed with *T. guianensis* seed extracts (5 to 30 µg GAE/mL) or PBS (negative control) for 20 min of incubation (37 °C, 100 revolutions per minute [RPM]). Then, AAPH (200 mmol/L) was added, allowed to react for 2 h (37 °C, 100 RPM) to complete the oxidation, and centrifuged at 1200 RPM for 10 min.

The hemolysis rate (%) was accessed using 100 µL of supernatant, mixed with 200 µL of PBS in a 96-well microplate, and recorded at 523 nm in a microplate reader. Regarding intracellular ROS generation, 400 µL of DCFH-DA solution (10 µmol/L) was mixed with the precipitate, incubated for 20 min at 37 °C in the dark, and transferred to a 96-well microplate to determine the fluorescence intensity at 485 and 520 nm for excitation and emission, respectively.

### 2.5. In Vitro Antimalarial Properties

The in vitro antimalarial effect of *T. guianensis* seed extracts was evaluated against W2 (chloroquine-resistant) and 3D7 (chloroquine-sensitive) strains. *P. falciparum* strains were cultivated in RPMI culture medium supplemented with 10% Albumax II and 4% hematocrit. The plates were incubated at 37 °C using the candle jar method [24]. The culture medium was replaced three times a week, and parasitemia was monitored using Panoptic fast-stained smears. Parasite synchronization at ring stages was performed with a 5% (*w*/*v*) D-sorbitol solution [25].

To perform the assay, the parasites were diluted to a solution with 1% parasitemia and 2% hematocrit and incubated on 96-well plates with different concentrations of *T. guianensis* seed extracts (0.2 to 12 μg GAE/mL) or with culture medium as a positive control. A 2% hematocrit solution was used as the negative control. After 48 h, 100 μL of parasite culture was added to a 96-well cell plate containing 100 μL of SYBR Gold at 1/10,000 (*v*/*v*) in lysis buffer [20 mM Tris, pH 7.5; 5 mM EDTA; 0.008% saponin (*w*/*v*); 0.08% Triton X-100 (*v*/*v*)]. The microplates were read using a microplate reader (excitation at 485 nm and emission at 535 nm). The selective index (SI) was calculated by the ratio between the IC_50_ normal cell (HUVEC) and the IC_50_ of each *P. falciparum* strain (3D7 and W2) [26].

### 2.6. Statistical Analysis

The experiments were performed in quadruplicate with results expressed as average ± SD. The dose–response analysis was determined by nonlinear regression (curve fit). The one-way analysis of variance (ANOVA) followed by the Tukey test was performed to investigate the differences among groups. Statistical analysis was performed using GraphPad Prism^®^ software (version 8.0, USA). Results with a *p*-value ≤ 0.05 were considered statistically significant.

## 3. Results and Discussion

### 3.1. Chemical Profile

Sample analyses revealed that the seed extracts contained 14–53 mg GAE/g TPC, and the equivolumetric mixture obtained from the water/ethanol extract (HE50) exhibited the highest phenolic content (Table 1), which is similar to that of mango seed extracts obtained by ultrasonic extraction (31–122 (±18) mg GAE/g) with the highest value obtained for an ethanol/water mixture [27]. Interestingly, the lowest phenolic content was observed for the ethanolic extract. Similar behavior was observed for the flavonoids (Table 1), where the ethanolic extract (EE100) exhibited the lowest TFC content (32 ± 4 mg CE/g) while the hydroethanolic extract (HE50) contained 158 ± 11 mg CE/g.

Regarding the total flavonol content (4–16 mg QE/g), no differences were observed between the amounts determined for the EE100, AE100, and HE80 extracts. In contrast, HE50 exhibited the highest total flavonol content; it also contained a higher ortho-diphenol content (33 mg CAE/g) than the other extracts (7–15 mg CAE/g). These findings are consistent with those of previous studies into hydroethanolic mixtures as optimal solvents for extracting phenolic compounds [22,28]. Castañeda-Valbuena et al. [26] suggested that lower ethanol concentrations (<80%) afford higher polyphenol contents from samples, consistent with our findings.

Overall, parameters such as temperature, solvent, contact area, time, molecular structure of the matrix, and the part of the plant used affect the extracted phenolic content [22]. Generally, hydroethanolic mixtures improve compound extraction efficiency and play fundamental roles in their solubilization, which is ascribable to the ability of water to swell the raw material, thereby facilitating the entry of ethanol into the cells. In contrast, protic ethanol is of lower polarity and enhances polyphenol solubilization, thereby improving the extraction efficiencies of hydrophilic and hydrophobic phenolics [27,29].

**Figure 1:**
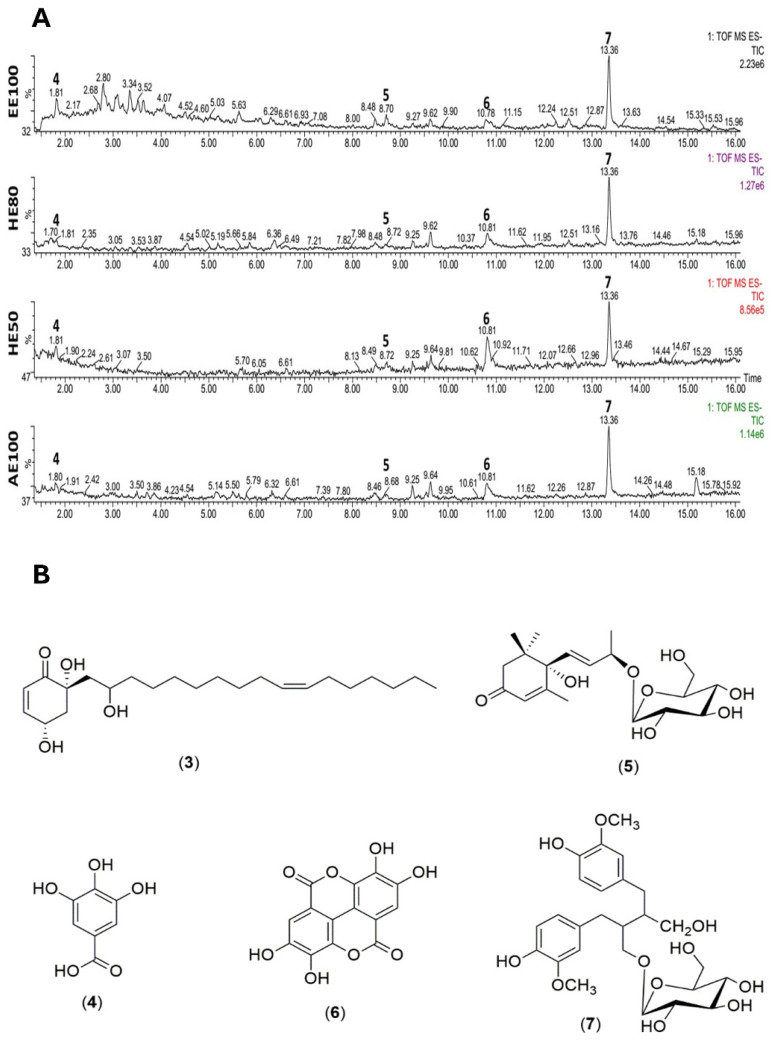
Chemical components of *T. guianensis* seed extracts. (**A**) UPLC-QToF/MS (negative mode) total ion chromatograms (TIC) of the four extracts: ethanolic extract (EE100), hydroethanolic extracts (HE80 and HE50), and aqueous extract (AE100). (**B**) Chemical structures of key compounds identified in *T. guianensis* seeds: (**3**) 4,6,2′-trihydroxy-6-[10′(Z)-heptadecenyl]-1-cyclohexene-2-one, (**4**) gallic acid, (**5**) (6S,7E,9S)-6,9-dihydroxy-megastigma-4,7-dien-3-one 9-*O*-β-glucopyranoside, (**6**) ellagic acid, and (**7**) (−)-Secoisolariciresinol-9′-*O*-β-d-glucopyranoside. The chromatograms illustrate the diversity of chemical profiles among the different extracts.

The chemical composition of the *T. guianensis* seed extracts was further explored by UPLC-QTOF/MS with the aim of examining the qualitative differences in composition between the four extracts. Figure 1A shows that the chromatographic profiles of the extracts are similar, with all samples exhibiting a main peak at 13.36 min, followed by a peak at 12.82 min. The constituents were identified by comparing 1D and 2D NMR data with those found in the literature (Table 2).

Additionally, compound **4**, which was proposed to be gallic acid (retention time = 1.8 min), exhibited an [M−H]^−^ ion at *m*/*z* 169 and an intense [M–H–CO_2_]^−^ ion at *m*/*z* 125 upon fragmentation (MS^2^), consistent with the loss of a carbon dioxide moiety. Compound **5** exhibited an ion at *m*/*z* 431 consistent with an [M+formic acid−H]^−^ adduct, a fragment at *m*/*z* 223 due to the loss of a hexose moiety, an [M−H−162]^−^ ion, and a peak at *m*/*z* 205 ascribable to the loss of a water molecule (i.e., [M−H−162−18]^−^). Compound **6** eluted at 10.8 min, presented an [M−H]^−^ ion at *m*/*z* 300.99, and was proposed to be ellagic acid (C_14_H_5_O_8_). Fragments (MS^2^) were observed at *m*/*z* 283 ([M−H−OH]^−^), 229 ([M−H−CO_2_−CO]^−^), and 185 ([M−H−2CO_2_−CO]^−^); this fragmentation pattern is similar to that reported by Ma et al. [33]. Compound **7** eluted at 13.3 min, presented peaks at *m*/*z* 523 (100% intensity, [M−H]^−^ ion) and 361.1647 ([M−glucose−H]^−^ ion), which are consistent with those previously reported for secoisolariciresinol monoglucoside (SMG) [34]. In addition, signals consistent with several carbohydrates, namely α- and β-glucose (**1**, **2**), 4,6,2′-trihydroxi-6-[10′(*Z*)-heptadecenyl]-1-cyclohexene-2-one (**3**), gallic acid (**4**), (6*S*,7*E*,9*S*)-6,9-dihydroxy-megastigma-4,7-dien-3-one 9-O-β-glucopyranoside (**5**), ellagic acid (**6**), and (−)-secoisolariciresinol-9′-*O*-β-d-glucopyranoside (**7**) were observed by 1D and 2D NMR spectroscopy (Figure 1B and Table 2).

The ^1^H NMR spectra revealed more intense signals at δ 7.53 (s) and δ 6.63 (d, J = 8.0 Hz, H-6) that correspond to constituents **6** and **7**, respectively, consistent with the LC findings, which identified these as the major constituents. While compounds **3**–**6** have been previously reported for *T. guianensis* [4,32,35], compound **7** has not been identified previously in this species.

### 3.2. Chemical Antioxidant Capacity of T. guianensis Seed Extracts

The antioxidant capacities of the four extracts were assessed using the DPPH, FRAP, and hydroxyl radical-scavenging assays, the results of which are shown in Table 1. The DPPH assay revealed that AE100 (119 mg AAE/g) was the most active, followed by HE50 (103 mg AAE/g). Similar behavior was observed for the hydroxyl radical-scavenging activity, with the aqueous extract (53 mg GAE/g) exhibiting notably higher activity than the other extracts.

HE50 and HE80 exhibited greater FRAP values than EE100 and AE100, which highlights the role played by the equivolumetric mixture of solvents in facilitating bioactive-compound extraction. These results are comparable with those reported by Do Carmo et al. [22] for *Myrciaria dubia* (camu-camu) seed extracts, in which the hydroethanolic extracts were also more active than the ethanolic and aqueous extracts. Moreover, HE50 and HE80 exhibited the highest TPC, TFC, and contents of flavonols and ortho-diphenols, which are known antioxidants [19].

The FRAP assay operates primarily through an electron-transfer mechanism and measures the ability of an antioxidant to reduce ferric (Fe^3^⁺) to ferrous (Fe^2^⁺) ions. The hydroxyl radical-scavenging assay, on the other hand, evaluates the capacity of an antioxidant to neutralize hydroxyl radicals (•OH) via hydrogen atom transfer, a critical mechanism for mitigating oxidative damage caused by ROS. In contrast, the DPPH assay is versatile as it measures antioxidant activity through both mechanisms, depending on the chemical structures of the bioactive compounds present in the sample [36]. The aqueous extract exhibited superior performance in the DPPH and hydroxyl radical assays, which is likely due to its hydrophilic antioxidant content, which includes phenolic acids (ellagic and gallic acid) that excel in these specific mechanisms. Conversely, HE50 exhibited greater efficacy in the FRAP assay, which suggests that it contained bioactive compounds with strong electron-donating capacities, and is possibly ascribable to moderately polar phenolic compounds.

### 3.3. Protection Against Lipid Peroxidation

**Figure 2:**
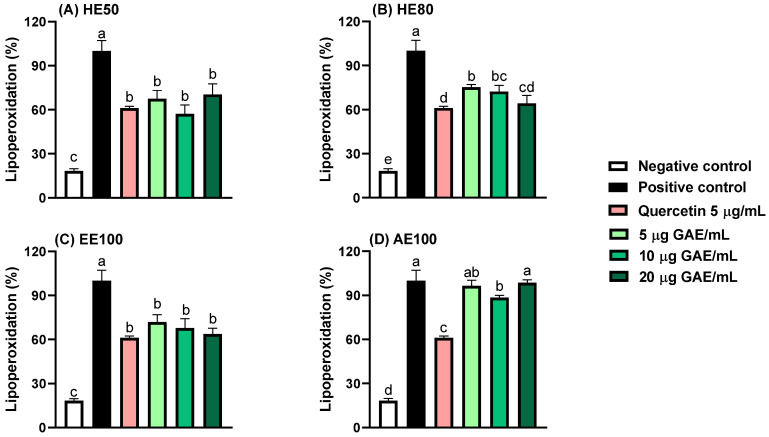
Effects of *T. guianensis* seed extracts on Fe^2^⁺-induced lipid peroxidation rates in egg yolk. (**A**) 50% hydroethanolic extract (HE50), (**B**) 80% hydroethanolic extract (HE80), (**C**) 100% ethanolic extract (EE100), and (**D**) 100% aqueous extract (AE100). Different letters within the same graph indicate statistically significant differences (*p* ≤ 0.05).

Lipid peroxidation rates were diminished by HE50, HE80, and EE100 at every tested concentration (5, 10, and 20 μg GAE/mL) (Figure 2). While HE50 and EE100 protected the lipids at the three tested concentrations with the same efficiency as quercetin, HE80 only exhibited standard effectiveness at 20 μg GAE/mL. Curiously, AE100 only effectively reduced the lipid peroxidation rate at 10 μg GAE/mL, despite being less efficient than the other samples; this is possibly ascribable to sample polarity, since extracts obtained using ethanol-rich solvents usually contain compounds that adequately interact with lipids to protect them from oxidative stress, while the high polarity of the aqueous extract may prevent it from reducing the lipid peroxidation rate [28]. Hydroethanolic *Myrciaria dubia* (camu-camu) seed extracts have been reported to inhibit lipid peroxidation by 24–86% [15], whereas optimized *Myrciaria cauliflora* (jaboticaba, currently reclassified as *Plinia cauliflora*) seed extracts reportedly inhibit lipid peroxidation by 71–86% [21], consistent with the results of the present study.

### 3.4. Cytotoxicity in Cell Culture

**Figure 3:**
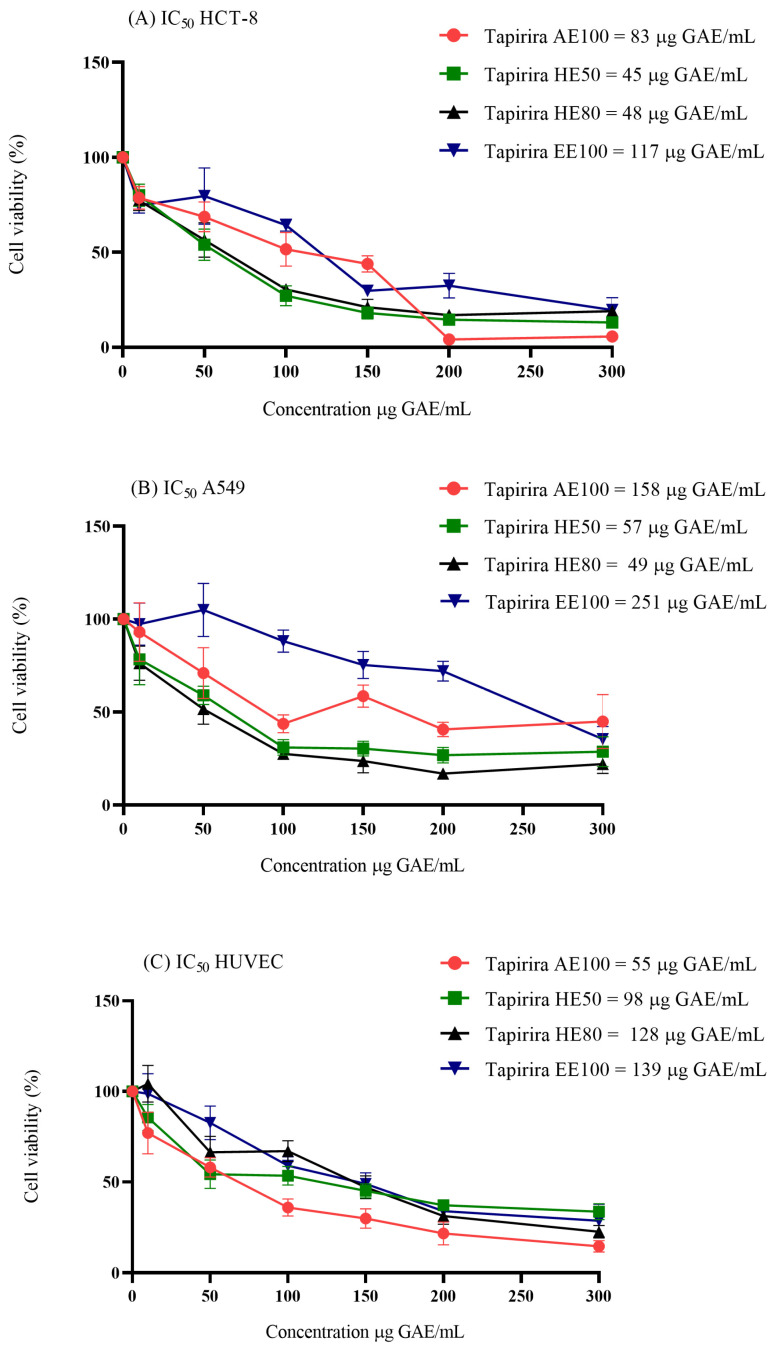
Cell viability and evaluation of the concentration-dependent effect after 48 h exposure to hydroethanolic extracts (HE50 and HE80), ethanolic extract (EE100), and aqueous extract (AE100) of *T. guianensis* seeds in (**A**) HCT-8, (**B**) A549, and (**C**) HUVEC cell lines. The IC_50_ (concentration of the agent that inhibits cell growth by 50%) values are indicated for each condition. Concentrations are expressed in μg GAE/mL of extract.

The relative cytotoxic potential of *T. guianensis* seed extracts was evaluated by comparing their performance against non-cancerous cells (HUVEC) and cancer cells (A549 and HCT-8), which revealed that all extracts are cytotoxic toward the examined cell lines (Figure 3). Among the tested cancer cells, A549 was least affected by EE100 and AE100, with IC_50_ values of 251 and 158 μg GAE/mL, respectively. These values are approximately twice as high as those observed for the HCT-8 cells (IC_50_ = 117 and 83 μg GAE/mL). The inner bark extract of *T. guianensis* reportedly exhibited similar behavior, with potent activity toward colorectal adenocarcinoma (CACO-2), carcinoma of the exocrine pancreas (PANC-1), and lung adenocarcinoma (CALU-6) cells, but lower sensitivity toward A549 cells [37]. Considering that SI values higher than three are indicative of selectivity toward cancer cells [16], the *T. guianensis* samples are not selective for cancer cells because their SI values ranged between 0.3 and 2.7 (Table 3).

The limited selectivity of these extracts (SI < 3) is possibly attributable to the presence of nonspecific cytotoxic compounds or insufficient molecular affinities for the unique characteristics of cancer cells. Further studies that focus on isolating the active fractions or combinations of extracts with targeted therapies may enhance their specificities. Moreover, the observed differences in IC_50_ underscore the need to investigate the molecular mechanisms responsible for the resistance of A549 cells compared to HCT-8, such as efflux pump activity or apoptotic pathway differences.

Among the samples, the 50% and 80% hydroethanolic extracts of *T. guianensis* were the most sensitive towards cancer cells, consistent with the findings of Silva-Oliveira et al. [38], who reported that the hydroethanolic *T. guianensis* leaf extract fraction was more cytotoxic than other fractions toward three head and neck cancer cell lines. Interestingly, a similar pattern was observed for camu-camu (*Myrciaria dubia*) seeds, in which a 50% hydroethanolic extract was found to be highly cytotoxic and selective toward A549 cells [22]. Overall, solvent polarity is particularly critical, as it significantly determines the phenolic compound compositional profile and its antioxidant activity. Solvents with medium and low polarities are capable of extracting compounds that are more able to permeate cell membranes and affect cells [29,39], which may explain the differences in cytotoxicity observed between the extracts.

With the exception of EE100 (A549), which is weakly active (IC_50_ = 201–500 μg/mL), the *T. guianensis* seed extracts are moderately active (IC_50_ = 21–200 μg/mL) according to the US National Cancer Institute (NCI), which classifies potential antiproliferative agents based on their cytotoxicity [14]. The presence of gallic and ellagic acid in the *T. guianensis* seed extracts may contribute to the cytotoxicity observed in the evaluated cells. Studies have shown that ellagic acid inhibits phosphorylation in the phosphatidylinositol 3-kinase (PI3K) and protein kinase B (Akt) signaling pathways, thereby promoting apoptosis by increasing the levels of p21, Bax, cytochrome c, caspase-3, and caspase-9, while reducing Bcl-2 and cyclin D1 levels in A549 cells [40,41]. Similarly, gallic acid suppresses the PI3K/Akt pathway and induces apoptosis by upregulating pro-apoptotic proteins such as Bax and caspase-3 [42]. These findings suggest that both ellagic and gallic acid are cytotoxic by modulating key signaling pathways associated with cell survival and apoptosis, as reflected by the activities of HE50 and HE80 in the A549 cells.

### 3.5. Intracellular Generation of Reactive Oxygen Species (ROS)

**Figure 4:**
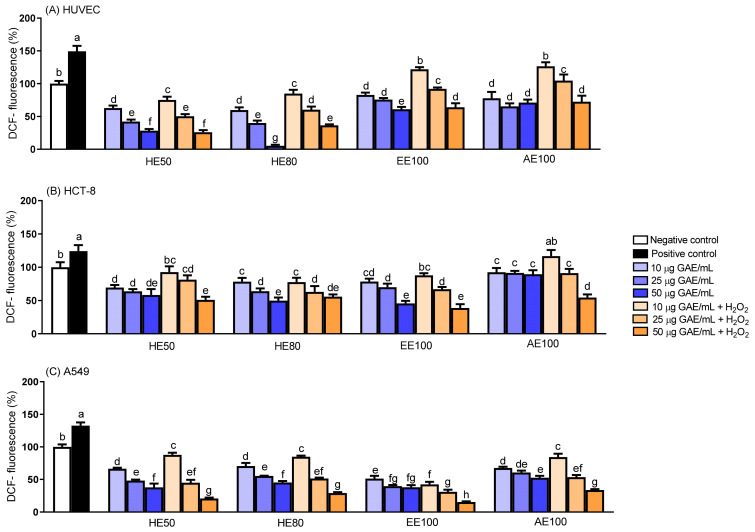
Intracellular ROS measurement in cell lines treated with *T. guianensis* extracts. Intracellular ROS levels were measured in (**A**) HUVEC, (**B**) HCT-8, and (**C**) A549 cells using a spectrofluorimetric method. The blue bars represent cells not challenged with H_2_O_2_, while the orange bars correspond to cells challenged with H_2_O_2_. The gradient of color intensity indicates different concentrations of the extracts (10 and 25 μg GAE/mL). Treatments included hydroethanolic extracts (HE50 and HE80), ethanolic extract (EE100), and aqueous extract (AE100) of *T. guianensis* seeds. Data are presented as the mean ± SD (n = 5). Different letters indicate statistically significant differences between treatments (*p* ≤ 0.05).

We used DCFH-DA to assess the ROS produced in cells exposed to various concentrations (10, 25, and 50 μg GAE/mL) of *T. guianensis* seed extracts. All extracts were found to decrease ROS generation in a concentration-dependent manner in the presence of H_2_O_2_, indicative of their cytoprotective capacities in cancer and non-cancer cells (Figure 4). Interestingly, the extracts reduced basal ROS production in non-cancerous cells (HUVEC) when treated in the absence of H_2_O_2_, particularly HE50 and HE80 at 50 μg GAE/mL, which lowered ROS levels by 70% and 95%, respectively. Although a pronounced reduction in ROS can disrupt cell signaling resulting in the loss of homeostasis control in normal cells [12], no detectable effect on cell proliferation was observed at these concentrations, which suggests that the observed reductions in ROS are likely due to localized antioxidant activities.

The ROS assay revealed that 50 μg GAE/mL HE80 or EE100 reduced ROS production in A549 cells by 55% or 58%, respectively, in the absence of H_2_O_2_. However, despite similar reductions in ROS levels, the A549 cells were more sensitive to the HE80 extract in terms of cell viability. Accordingly, our results suggest that the concentration required to inhibit cell proliferation, as observed in the cell viability test, may involve mechanisms unrelated to ROS generation, as discussed in Section 3.2. Similar behavior was observed in studies involving *Vitis vinifera* and *Myrciaria dubia* seed extracts, where the cytotoxic effects toward cancer cells and ROS reduction were found not to correlate [22,43].

The phenolic compounds present in the *T. guianensis* seed extracts could be associated with the lower oxidative stress observed in the cells, with and without H_2_O_2_ induction. This antioxidative effect could be particularly linked to SMG, a diphenolic nonsteroidal phytoestrogen belonging to the lignan family [44], which has been identified as the predominant compound in all *T. guianensis* extracts (HE50, HE80, EE100, and AE100). The antioxidant properties of SMG are attributable to the hydroxyl groups at the para positions of its phenolic rings [45]. Similarly, other lignan-rich extracts, such as those from flaxseed [46], exhibit antioxidant activities that are strongly associated with lignans such as secoisolariciresinol diglucoside (SDG) that are similarly hydroxylated. This comparison underscores the potential of SMG as a key compound whose antioxidant properties and potential applications need to be further investigated.

Overall, antioxidant effects were observed for both cancer and non-cancer cells. ROS are produced in a wide variety of cellular processes under physiological conditions, and play vital roles in stimulating signaling pathways, such as intracellular signal transduction, metabolism, proliferation, and apoptosis in normal cells, as well as in regulating the immune system and maintaining redox balance [47,48]. In contrast, antioxidant/oxidant imbalance can lead to high ROS concentrations, potentially resulting in DNA mutations and malignant cell transformations [49,50]. Consequently, evaluating the antioxidant capacities of *T. guianensis* seed extracts underscores their potential protective effects against oxidative damage to the cells evaluated in this study.

### 3.6. Chromosomal Aberrations

The therapeutic potential of plant-derived extracts has been increasingly explored in cancer research, particularly for their abilities to mitigate genotoxic damage induced by chemotherapeutic agents [51]. The HE50 extract was chosen for chromosomal aberration testing owing to its chemical composition, which is rich in phenolic compounds, flavonoids, flavonols, and ortho-diphenols that are recognized as having antioxidant and cytoprotective properties [52,53]. To induce chromosomal aberration, a known alkylating agent (cisplatin) was used [22]. Cisplatin blocks cell division and leads to cancer-cell apoptosis by selectively binding to 1,2-intra- and inter-strand crosslinks involving the purine bases in DNA, thereby disrupting repair and replication. Apoptosis also involves intrinsic and extrinsic mitochondrial pathways, such as p53 signaling and cell cycle arrest, which upregulate pro-apoptotic genes and proteins [47,51].

Table 4 shows that HE50 on its own did not induce chromosomal aberrations in A549 cells, even at 20 µg GAE/mL, nor did it reduce chromosomal damage to 50% below basal levels at 50 µg GAE/mL. Furthermore, 5, 10, and 20 µg GAE/mL of the extract reduced chromosomal aberrations caused by cisplatin by 32%, 43%, and 45%, respectively. Similarly, another study observed that camu-camu seed extract is protective, decreasing the chromosomal break index by 37% and attenuating cisplatin-induced mutagenic damage [22]. Cisplatin has been shown to induce lipid peroxidation and ROS directly or indirectly through mitochondria, thereby impairing the synthesis of electron transport chain proteins [47,51]. Taken together, the lower lipid peroxidation rate, ROS generation, and A549 cell viability suggest that our extract is able to inhibit chromosomal aberrations and is protective against cisplatin-induced oxidative damage, which is likely ascribable to its antioxidant and cytotoxic properties [22], as demonstrated in the previous sections.

**Figure 5:**
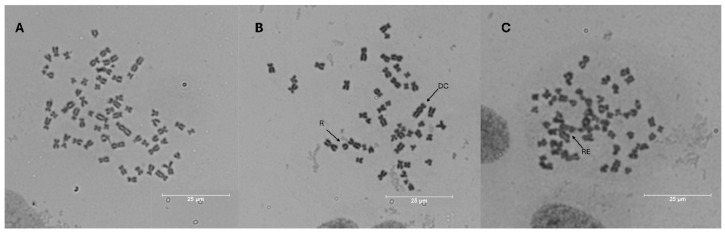
Photomicrographs (1000×) of metaphase plates of A549 cells. (**A**) Negative control, (**B**) 4 μM cisplatin and (**C**) 20 µg GAE/mL group. Different kinds of chromosomal aberrations were observed, such as rings (R), dicentric chromosomes (DC), and rearrangements (RE).

Additionally, compared to basal events (Figure 5A), the chromosomal aberrations frequently observed in our study, such as dicentric chromosomes (CD), rearrangements (RE), and rings (R), are shown in Figure 5B,C. According to Bonassi et al. [54], a large number of rearrangements are indicative of intense genomic damage, and are often associated with severe genotoxicity and chromosomal instability, which suggests that cisplatin is profoundly damaging. Table 4 reveals that the HE50 extract reduced rearrangement events by more than 50% and protected A549 cells against cisplatin-induced damage by acting as an antimutagenic agent.

### 3.7. Erythrocyte Protection Effects of T. guianensis Extracts

**Figure 6:**
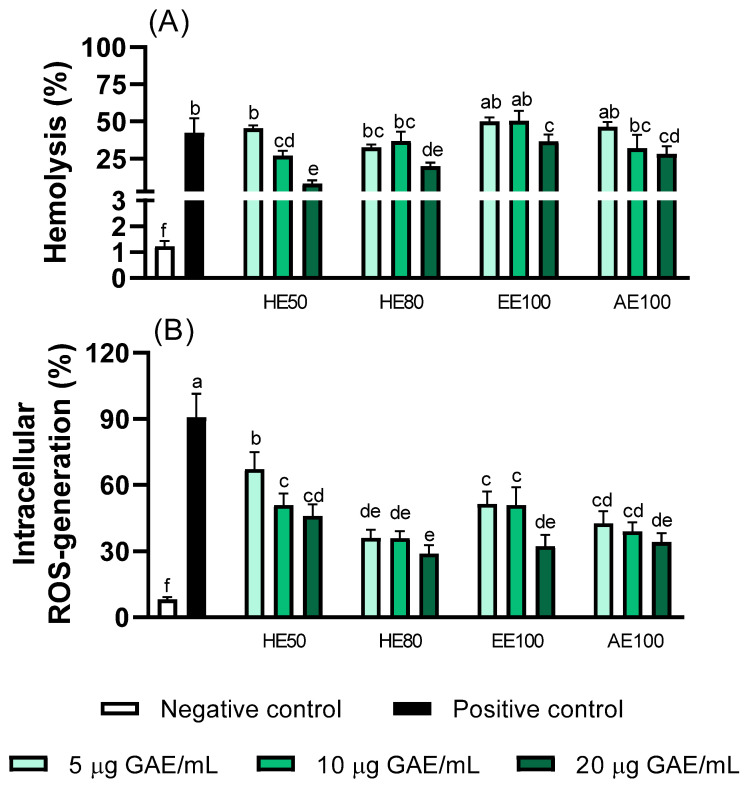
Effects of *T. guianensis* extracts on AAPH-induced hemolysis and intracellular ROS generation in human erythrocytes. (**A**) AAPH-induced hemolysis in human erythrocytes. (**B**) Intracellular ROS generation following AAPH treatment. Different letters indicate statistically significant differences between treatments (*p* ≤ 0.05).

Hemolysis rate (%) and intracellular ROS were measured to evaluate the abilities of the *T. guianensis* extracts to protect red blood cells (RBCs) from AAPH-induced oxidative stress (Figure 6). Considering that RBCs are vulnerable to oxidative damage owing to redox reactions involving hemoglobin [23], extracts with higher concentrations of phenolic compounds are expected to present various degrees of antioxidant activity that reflect reduced levels of ROS and a less oxidative environment [12].

All extracts were anti-hemolytic and therefore able to protect the erythrocyte membrane from lysis (Figure 6A). The HE50 extract significantly reduced the hemolytic response toward AAPH-induced stress in a dose-dependent manner, whereas lower levels of hemolysis were observed for HE80, EE100, and AE100 at higher concentrations. Intracellular ROS exhibited the same behavior, with the HE80 extract providing the greatest dose-dependent protection against AAPH-induced oxidation (67% reduction at 20 µg GAE/mL) compared to the HE50 and AE100 extracts (Figure 6B). In summary, the hydroethanolic extracts (HE50 and HE80) were found to offer greater antioxidant protection in erythrocytes than the other extracts.

These findings are consistent with those of previous studies in which certain polyphenol-rich natural extracts were shown to have antioxidant activities and reduce the levels of intracellular ROS and RBC hemolysis [14,23].

Literature reports on natural extracts have shown that the above-mentioned membrane protection and response are mainly attributable to the extraction type and phenolic content, as demonstrated by our chemical analysis that provides further evidence of this relationship [19,23]. In addition, our results using HE50 and HE80 reveal that an appropriate hydrophilic/lipophilic polyphenol balance, which is achieved by considering the extraction type, effectively extracts compounds that suppress ROS and stabilizes membranes without excessive disruption [19].

### 3.8. T. guianensis Extracts Present Antimalarial Activity Against P. falciparum

The efficacy of antimalarial drugs toward blood-stage infection has been increasingly threatened since the spread of chloroquine resistance in *Plasmodium falciparum* in 1960 [55,56]. This widespread resistance underscores the urgent need to identify new antimalarial agents capable of acting against both chloroquine-sensitive and chloroquine-resistant strains to ensure global applicability and effectiveness.

**Figure 7:**
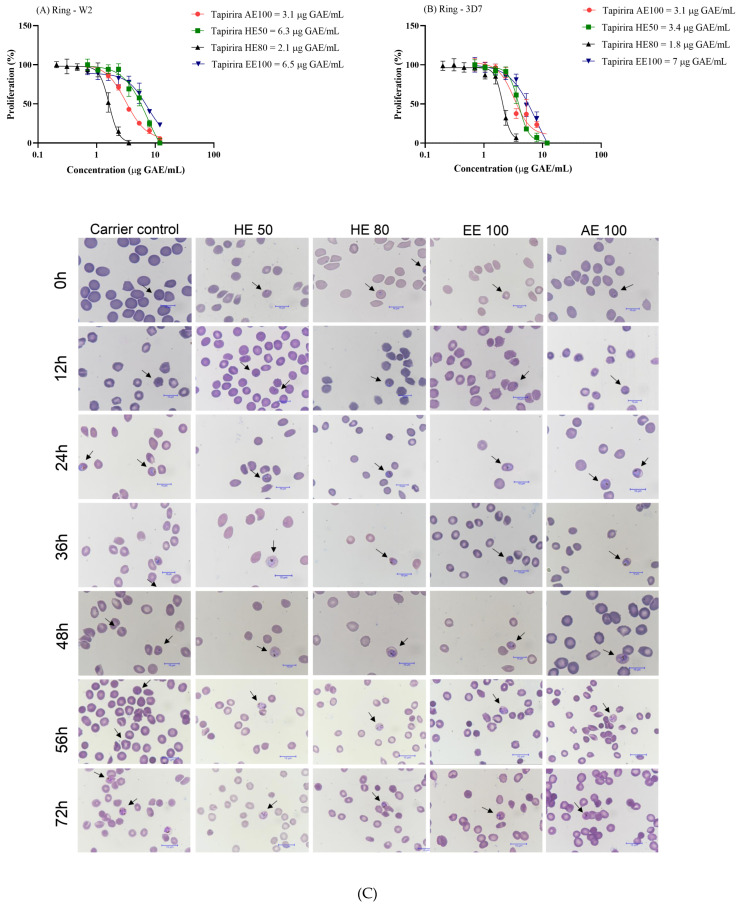
Antimalarial activity and dose–response effects of *T. guianensis* seed extracts on *P. falciparum*. (**A**) Dose–response curves and IC_50_ values for hydroethanolic extracts (HE50 and HE80), ethanolic extract (EE100), and aqueous extract (AE100) against the W2 chloroquine-resistant strain of *P. falciparum*. (**B**) Dose–response curves and IC_50_ values of the same extracts against the 3D7 chloroquine-sensitive strain. (**C**) Microscopy images of panoptic fast-stained smears showing tightly synchronized *P. falciparum* 3D7 strain at different intervals. Arrows indicate intraerythrocytic structures in cultures treated with RPMI medium containing 10% Albumax II (control) or the extracts. Concentrations are expressed in μg GAE/mL of seed extract.

The antiplasmodial activity of *T. guianensis* seed extracts was assessed against both the 3D7 (chloroquine-sensitive) and W2 (chloroquine-resistant) strains of *P. falciparum*. Remarkably, both strains exhibited high sensitivity to all tested extracts (Figure 7A,B). Considering the antiparasitic activity classification system introduced by Jansen et al. [57], HE80 and AE100 exhibited the lowest IC_50_ concentrations and high antimalarial activities toward both strains (IC_50_ ≤ 5 μg/mL), while HE50 and EE100 demonstrated promising activities (5 μg/mL < IC_50_ ≤ 15 μg/mL). These results highlight the potential of *T. guianensis* extracts for overcoming the challenges posed by chloroquine resistance, which is prevalent in many endemic regions [58,59].

Roumy et al. [4] evaluated the anti-plasmodial activity of a dichloromethane extract of *T. guianensis* bark and its isolated fractions. A mixture of 4,6,20-trihydroxy-6-[100(*Z*)-heptadecenyl]-2-cyclohexenone and 1,4,6-trihydroxy-1,20-epoxy-6-[100(*Z*)-heptadecenyl]-2-cyclohexene delivered IC_50_ values against chloroquine-sensitive and chloroquine-resistant strains that were four to five times lower than those of the dichloromethanolic extract. This promising activity against *P. falciparum* is attributable to 4,6,20-trihydroxy-6-[100(*Z*)-heptadecenyl]-2-cyclohexanone, which contains a conjugated ketone, a feature often associated with pharmacological activity. The same compound was found in the extracts of *T. guianensis* seeds, and may serve as an accessible therapeutic alternative anti-plasmodial drug for further investigation.

Evaluating SI values is crucial for identifying promising non-toxic and safe antimalarial agents; SI > 10 is considered indicative of a lack of cellular toxicity compared to that of *P. falciparum* [26,60]. This parameter is especially important for ensuring that a treatment selectively targets the parasite while preserving human cells. In this study, all the extracts exhibited SI values greater than ten for both strains (SI: 15.5–70.5). In particular, the SI value for HE80 indicates that this *T. guianensis* seed extract is significantly more toxic toward *P. falciparum* than HUVEC.

To determine how the *T. guianensis* seed extracts act against *Plasmodium falciparum*, we investigated which stage of the parasite’s intraerythrocytic cycle is affected. Cultures starting at 1% parasitemia (3D7 strain) and 2% hematocrit were treated with an IC_50_ concentration of each *T. guianensis* extract for 72 h. Panoptic fast-stained smears were prepared using infected erythrocytes from cultures in several moments of the cycle. Figure 7C shows that the *T. guianensis* extracts affected the first intracellular developmental cycle (IDC), thereby preventing parasite growth beyond the schizont stage and inhibiting schizont segmentation and erythrocyte rupturing. This effect on the first IDC is aligned with the recommendations from the World Health Organization [8], as it mirrors the action of artemisinin, the reference drug in antimalarial treatment.

Additionally, the observed effect is superior to those of common antimalarial drugs, such as clindamycin, doxycycline, and atovaquone that exhibit delayed death by targeting apicoplast replication [61] or ubiquinone metabolism [62] in later IDCs. On the other hand, drugs that affect essential apicoplast pathways, such as those involved in fatty acid or isoprenoid biosynthesis, are particularly valuable as they inhibit the parasite during the first IDC [63,64]. The ability of the *T. guianensis* seed extracts to kill *P. falciparum* in the first IDC suggests that they contain bioactive compounds with antimalarial potential that may target similar pathways or act on complementary mechanisms to those of fast-acting drugs, such as artemisinin. Future studies should investigate whether or not any components of the *T. guianensis* act as inhibitors of these metabolic pathways, thereby providing new insight into its mechanism of action and potential as a source of novel targeted antimalarial agents.

Fast-acting drugs such as artemisinin and its derivatives play critical malaria treatment roles by rapidly reducing parasitemia and preventing disease progression, especially in severe cases. The fact that the extract in this study demonstrates comparable effects is highly relevant given (i) the limited number of rapidly acting antimalarials and (ii) the increasing need for new drugs to address resistance and treatment efficacy. Additionally, the development of compounds with complementary or synergistic targets to fast-acting drugs could enhance therapeutic efficacy and delay resistance emergence [8], which reinforces the need to characterize the active constituents of *T. guianensis* and elucidate their mechanisms of action.

## 4. Conclusions

In this study, we showed that *T. guianensis* seeds have active constituents with promising therapeutic potential supported by the cytoprotective, antiproliferative, and antimalarial activities of hydroethanolic extracts. These findings, along with its chemical profile and antioxidant capacity, highlight *T. guianensis* as a valuable source of bioactive compounds. The HE50 and HE80 extracts were cytotoxic toward cancer cells compared to normal cells, which reinforces their potential as a source of selective anti-proliferative agents. In addition, the extracts exhibited robust antioxidant properties and reduced the levels of reactive oxygen species (ROS) generated in cancer and normal cells, while protecting erythrocytes against oxidative damage. The ability of HE50 to mitigate cisplatin-induced chromosomal aberrations highlights its cytoprotective and antimutagenic potential. Similarly, the HE80 extract exhibited high selectivity indices, especially in antimalarial assays, with SI values of 70 for the chloroquine-sensitive strain of *Plasmodium falciparum* (3D7) and 60 for the chloroquine-resistant strain (W2); hence, HE80 exhibits highly specific toxicity toward these parasites. These results highlight the therapeutic relevance of compounds extracted from *T. guianensis* seeds, particularly when extracted with hydroethanolic solvents, and provide a pathway for future in vivo safety and efficacy studies.

## Figures and Tables

**Table 1:** Phenolic compound concentrations and antioxidant capacities per gram of dried *T. guianensis* seed extracts. Antioxidant activity is expressed in units consistent with standard reporting practices (e.g., mg AAE/g or mg GAE/g), where higher values indicate greater antioxidant capacity. Data are presented as mean ± standard deviation (n = X), and different superscript letters indicate statistically significant differences (*p* ≤ 0.05).

Compounds	HE50	HE80	EE100	AE100
TPC (mg GAE/g)	53 ± 1 ^a^	23 ± 1 ^c^	14 ± 0.6 ^d^	32 ± 0.7 ^b^
TFC (mg CE/g)	158 ± 11 ^a^	113 ± 2 ^c^	32 ± 4 ^d^	132 ± 2 ^b^
Total flavonol content (mg QE/g)	16 ± 1 ^a^	4 ± 0.3 ^b^	6 ± 0.7 ^b^	5 ± 1 ^b^
Ortho-diphenols (mg CAE/g)	33 ± 1 ^a^	15 ± 0.5 ^b^	7 ± 0.4 ^c^	15 ± 1 ^b^
**Antioxidant activity**				
DPPH (mg AAE/g)	103 ± 3 ^b^	78 ± 1 ^c^	27 ± 2 ^d^	119 ± 1 ^a^
FRAP (mg AAE/g)	87 ± 2 ^a^	54 ± 1 ^b^	24 ± 1 ^d^	50 ± 0.3 ^c^
Hydroxyl radical-scavenging activity (mg GAE/g)	16 ± 0.4 ^d^	44 ± 1 ^b^	23 ± 0.5 ^c^	53 ± 2 ^a^

Note: TPC, total phenolic content; TFC, total flavonoid content; DPPH, 2,2-diphenyl-1-picrylhydrazyl; FRAP, ferric reducing antioxidant power; GAE, gallic acid equivalent; CE, catechin equivalent; QE, quercetin equivalent; CAE, chlorogenic acid equivalent; AAE, ascorbic acid equivalent; HE50 and HE80 hydroethanolic extracts (50% and 80% of ethanol), EE100 (ethanolic extract), and EA100 (aqueous extract). Different letters in the same row represent significant differences (*p* ≤ 0.05).

**Table 2:** Compounds identified in the dried *T. guianensis* seed extracts by using UPLC-ESI-QTOF-MS/MS (negative mode) and 1D and 2D NMR.

No	Retention Time	Adduct	*m*/*z*	Identified Mass	Calculated Mass	Fragmentations (*m*/*z*)	Compound (Empirical Formula, Error in ppm)	δ ^1^H in ppm (*J*, Hz)	δ ^13^C in ppm	References
1	-	-	-	-	-	-	α-glucose	δ 5.08 (d; *J* = 3.7 Hz, H-1)	93.8 (C-1), 71.8 (C-2), 73.5 (C3).	[30]
2	-	-	-	-	-	-	β-glucose	δ 4.45 (d; *J* = 7.7 Hz, H-1) δ 3.31 (dd; *J* = 9.2; 7.8 Hz, H-3)	98.0 (C-1), 77.8 (C-3), 70.3 (C-4).	[30]
3	-	-	-	-	-	-	4,6,2′-trihydroxi-6-[10′(Z)-heptadecenyl]-1-cyclohexene-2-one (C_23_H_40_O_4_)	5.88 (dd, *J* = 10.2; 2.0 Hz, H-2); 6.92 (m, H-3); 1.90 (m, H-1′), 4.02 (m, H-2′); 5.31 (t, *J* = 5.0 Hz, H-10′, H-11′); 2.0 (m, H-12′), 0.88 (t, *J* = 7.0 Hz, H-17′).	126.2 (C-2), 153.6 (C-3), 64.7 (C-4), 43.3 (C-1′), 70.7 (C-2′), 130.5 (C10′, C11′), 27.8 (C12′), 14.0 (C-17′).	[4]
4	1.8	[M−H]^−^	169.0133	170.0211	170.0215	125.0245	Gallic acid (C_7_H_5_O_5_, -2.35)	7.03 (s, H-2,6)	108.8 (C-2, 6), 145.0 (C-3,5), 138.5 (C-4), 168.8 (C-7).	[1,31]
5	8.7	[M+formic acid-H]^-^	385.1892	386.1970	386.1940	431.1931 223.1338 205.1258	(6*S*,7*E*,9*S*)-6,9-dihydroxy-megastigma-4,7-dien-3-one 9-*O*-β-glucopyranoside (C_19_H_30_O_8_, 7.77)	2.44 (m, H-2ax) 2.56 (m, H-2eq)	50.5 (C-2), 200.0 (C-3), 131.3 (C-7), 71.8 (C-4′).	[32]
6	10.8	[M−H]^−^	300.9990	302.00683	302.00626	283.9960 229.0141 185.0220	Ellagic acid (C_14_H_5_O_8_, 1.88)	7.53 (s, H-5′, H-5′).	113.0 (C-1, C-1′), 139.7 (C-3, C-3′), 148.0 (C-4, C-4′), 110.5 (C5, C-5′), 108.1 (C6, C-6′), 160.0 (C-7, C-7′).	[31,33]
7	13.3	[M−H]^−^	523.2188	524.2266	524.2257	361.1647	(−)-Secoisolariciresinol-9′-*O*-β-d-glucopyranoside (C_26_H_36_O_11_, 1.71)	6.56 (d, *J* = 1.8 Hz, H-2); 6.63 (d, *J* = 8.0 Hz, H-6); 3.49 (d, *J* = 5.1 Hz, H-9); 2.49–2.54 (m, H-7, H-7′); 1.91 (m, H-8); 6.58 (d, *J* = 1.8 Hz, H-2′); 6.63 (d, *J* = 8.0 Hz, H-5′); 6.53 (m, H-6′); 4.20 (d, *J* = 7.8 Hz, H-1′’); 3.84 (d, *J* = 1.8 Hz, H-6’’); 3.63 (m, H-6’’); 3.82 (m, OCH_3_-3); 3.71 (s, OCH_3_-3′).	113.3 (C-2), 147.2 (C-4), 122.4 (C-6), 61.1 (C9), 36.5 (C-7, C-7′); 43.5 (C-8), 132.7 (C-1′), 115.8 (C-2′), 147.4 (C-3′), 144.2 (C-4′), 115.5 (C-5′), 122.4 (C-6′), 104.3 (C-1″); 62.5 (C-6″), 56.4 (OCH_3_-3); 56.0 (OCH_3_-3′)	[34]

**Table 3:** Selectivity Index (SI) of *T. guianensis* extracts. SI values calculated as HUVEC IC_50_ divided by IC_50_ for *P. falciparum* strains 3D7 and W2, and cancer cell lines A549 and HCT-8. Higher SI indicates greater selectivity.

Extracts	HUVEC/*P. falciparum*		HUVEC/Cancer Cells	
	3D7	W2	A549	HCT-8
HE50	28.4	15.5	1.7	2.2
HE80	70.5	60.2	2.6	2.7
EE100	20	21.3	0.6	1.2
AE100	17.6	18	0.3	0.7

Note: Selectivity Index (SI) for cancer cells is calculated as IC_50_ HUVEC/IC_50_ cancer cells. Similarly, the Selectivity Index (SI) for *P. falciparum* is determined as IC_50_ HUVEC/IC_50_ *P. falciparum* strains. HE50 and HE80 hydroethanolic extracts (50% and 80% of ethanol), EE100 (ethanolic extract), and AE100 (aqueous extract).

**Table 4:** Results of the chromosome aberrations test in A549 cells treated with HE50 extract in vitro.

HE50 (µg GAE/mL)	CIS	TC	Aberrant Type								TNCA	CA Rate (%)
			R	DC	FR	CB	CEB	TC	QC	RE		
NC	-	4315	2	31	0	0	0	1	1	7	42 ^b^	1
PC	4 μM	5239	8	40	0	0	0	3	5	22	78 ^a^	1.5
20	-	4343	3	19	1	0	0	1	7	9	40 ^b^	1
50	-	4367	0	15	1	0	0	1	2	2	21 ^c^	0.5
5	4 μM	4045	7	29	1	1	1	7	2	6	41 ^b^	1
10	4 μM	4038	5	22	3	4	1	2	6	4	34 ^b^	1
20	4 μM	4269	3	30	3	1	1	2	4	4	35 ^bc^	1

Note: CIS: cisplatin; TC: total cells; R: rings; DC: dicentric chromosomes; FR: fragments; CB: chromatid breaks; CEB: chromosome breaks; TC: triradial chromosomes; QC: quadriradial chromosomes; RE: rearrangements; TNCA: total number of chromosome-aberrant cells; CA: chromosomal aberrations; NC: negative control; PC: positive control. In the presence of multiple types of chromosomal aberrations during the metaphase of a cell, the number of chromosomal aberrations was counted as 1. a–c indicates the significant difference when compared (*p* < 0.05).

## Data Availability

The original contributions presented in this study are included in the article. Further inquiries can be directed to the corresponding author.
